# Fostering Belonging Through Introducing Diverse Scientists in Undergraduate Neuroscience Lectures

**DOI:** 10.59390/001c.161078

**Published:** 2026-06-23

**Authors:** Katharine V. Northcutt

**Affiliations:** 1 Biology and Neuroscience Mercer University

**Keywords:** diversity, lecture, neuroscientists, belonging, representation

## Abstract

Fostering a sense of belonging in the scientific community is one way to increase the retention of students who belong to groups that have been historically excluded in STEM. In the past several years, I have attempted this in my undergraduate Neurobiology course by introducing students to several neuroscientists with each topic we cover. For each scientist, I spend 2-5 minutes total giving biographical information, introducing an example of the type of research they do, and explaining how their research is related to the lecture topic. The neuroscientists I have included are diverse in racial/ethnic background, gender identity, nationality, sexual orientation, disabilities/abilities, career trajectory, and career stage. Based on an anonymous survey, students overwhelmingly appreciated this aspect of lectures, reported that they are more interested in course material, and were more aware of career options in neuroscience. Students belonging to a group that has been historically under-represented in the sciences were particularly likely to report that they felt more represented in science. Fifty-three percent reported that they are more likely to pursue a career related to neuroscience because of this component of the course. Students said that “meeting” neuroscientists helped them not only realize the diversity of neuroscientists themselves but also the diversity of sub-fields within neuroscience. They also felt more empowered to get involved in research because I included examples of scientists in earlier career stages, such as graduate students, that were more relatable. This is an easy way to make a course more inclusive and can be used in a wide variety of course formats and sizes. It is not time intensive, complements existing course topics, and has a positive impact on students’ classroom experience.

## Introduction

Despite the increasing diversity among college students, the retention and completion of STEM degrees is lower among historically under-represented groups (Chang et al., 2014; Hughes, 2018; Shaw & Barbuti, 2010). One of the factors that contributes to student success in individual courses is a sense of belonging, which includes feeling valued and legitimate in STEM settings (Cwik & Singh, 2022). Students from historically under-represented groups in STEM typically report less belonging (Barnes et al., 2021; Freeman, 2020; Lewis et al., 2017; Rainey et al., 2018), and belonging is one predictor of persistence in a STEM major (Hansen et al., 2024; Lewis et al., 2017; Rainey et al., 2018). Holmegaard et al. (2014) note that STEM identities are continuously evolving, and they can be affected by course and institutional policies, prevailing stereotypes, and the representation of identities of other students and faculty that students see around them (Harry et al., 2024; Lewis et al., 2017).

Developing an identity as a scientist is also an important predictor of college success and is positively correlated with choosing a career in the sciences (Chen et al., 2021; Trujillo & Tanner, 2014). Similar to belonging, identifying as a scientist is particularly important for success for under-represented minority students (Chen et al., 2021). Furthermore, a science identity and a sense of belonging affect one another — having a strong sense of belonging is an important step in developing an identity as a scientist, but identifying as a scientist can also help a student feel that they belong in a STEM classroom or laboratory.

Belonging and identity can affect the motivation and resilience that students have throughout college. For example, students who feel more belonging and STEM identity may view one low exam grade as a misstep in their journey, whereas those who are struggling to find their place in STEM may view this as confirmation that they are not suited for a STEM major or career (Chen et al., 2021). Although students from historically under-represented groups tend to report less belonging and sense of scientific identity, these students receive the most benefit from curricular and co-curricular experiences designed to help them see their place in STEM (Ho et al., 2026).

Although program-wide structures, such as opportunities for undergraduate research, can improve students’ belonging and identity (White et al., 2025), there are also numerous benefits from modifications within courses. For example, active learning can increase belonging and development of a scientific identity (Jiang & Wei, 2025). The ways that professors support students, both as students who are learning science but also as people with interests and concerns beyond the classroom, can also have a large impact on student belonging (Harry et al., 2024; Jiang & Wei, 2025; Rattan et al., 2018).

One additional way that we can increase belonging and science identity is through increasing the representation of scientists in our courses. In a recent study of marginalized college student experiences in the life sciences, students specifically mentioned a desire to see more representation in instructional materials, such as using anatomical models of multiple skin colors and assigning resources written by authors who are not all white males (Harry et al., 2024). This likely increases the support that under-represented students perceive from their instructors. Out-of-class assignments introducing students to a diverse group of scientists, including their research and their personal history as a scientist, through “Scientist Spotlights” have been shown to increase the degree to which students feel themselves represented in science and increase biology course grades (Aranda et al., 2021; Schinske et al., 2016; Yonas et al., 2020). These interventions have been particularly impactful at increasing relatability of scientists for first-generation students (Metzger et al., 2023). Many Scientist Spotlights (scientistspotlights.org) have been written by undergraduate students, and the experience of authoring these resources further bolstered students’ ability to see themselves as scientists (Aranda et al., 2021). Recently, Project DiViNE began efforts to make a database to share biographies of a diverse group of neuroscientists (Frenzel & Harrington, 2022). They published five of these biographies (about Rita Levi-Montalcini, Ricardo Miledi, Simon Levay, Erich Jarvis, and Steve Ramirez), with corresponding publications and teaching resources that could be used to teach about these neuroscientists in class (Frenzel et al., 2022). These are valuable resources for developing in- and out-of-class assignments that complement student learning about neuroscience concepts.

In addition to using these resources, I was interested in developing ways to briefly highlight diverse scientists throughout lectures, including through describing scientists who are currently at a wide variety of career stages. One of the stereotypes that students often hold about scientists is that they are “older” (Sheffield et al., 2021), and I hoped that including this additional aspect of diversity would increase the degree to which students relate to these scientists, thus furthering their development of belonging and science identity. As a result, I included short introductions to a wide variety of scientists throughout my 400-level Neurobiology course, and I assessed student perceptions of this aspect of the course.

## Materials and Methods

### Identifying neuroscientists to highlight

I began by creating a list of ideas of neuroscientists that I could highlight in lectures, which included 4-5 neuroscientists for each of the 14 topics I include in the course (Supplemental Table 1 lists these topics). These scientists are doing or have done research related to that topic. Throughout the list, I strived for having diversity in racial/ethnic background, gender identity, nationality, sexual orientation, disabilities/abilities, career trajectory, and career stage. I included some neuroscientists that I know personally, including friends and collaborators, fellow students I knew in graduate school, people I see occasionally at conferences, and my own former students. I found other ideas from searching 500 Queer Scientists (https://500queerscientists.com/), the Black in Neuro member directory (https://blackinneuro.com/), 100 Inspiring Hispanic/Latinx Scientists in America (https://crosstalk.cell.com/blog/100-inspiring-hispanic-latinx-scientists-in-america), and public-facing social media profiles. I also aimed to include scientists who have shared information about their personal interests or hobbies outside of science.

### Introducing neuroscientists during lectures

On the first day of class, I tell students that I will be including a diverse array of scientists during class. I explain why I am doing this, and I also let students know that I welcome feedback, particularly about this aspect of the class (they can always leave anonymous notes under my office door if they prefer not to be identified). I also include this “diversity statement” on the syllabus: “Science has primarily been done by a small set of privileged people. One of my favorite things about neuroscience is that there is a diverse array of people in the field, but some biases remain and scientists from historically excluded groups still face discrimination. One of my goals in this class is to introduce you to a wide range of neuroscientists in hopes that the field of neuroscience and the scientists who do this work become more relatable to you.”

**Figure 1.**
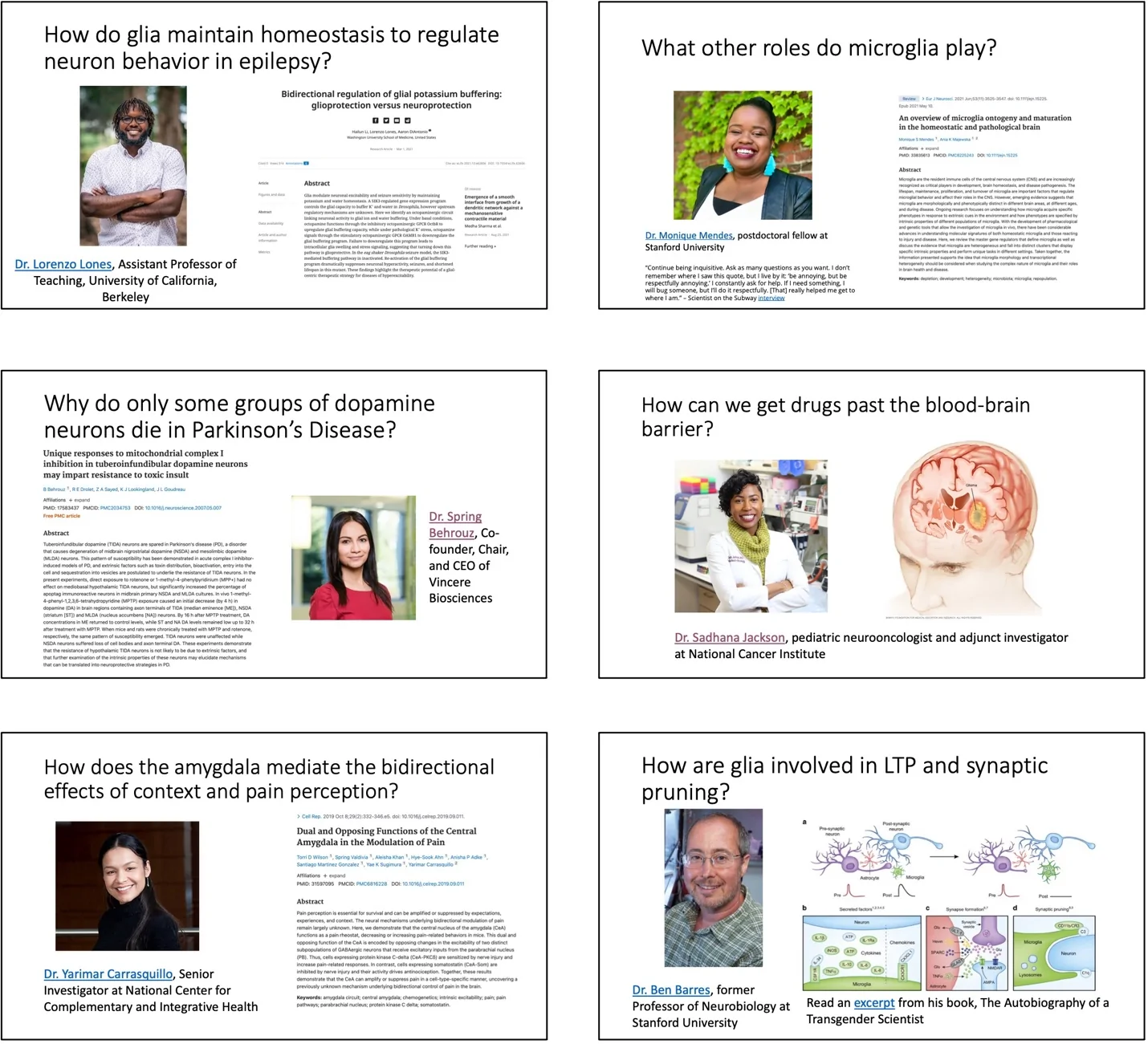
Example slides introducing scientists.

Each semester, I narrow down my list of scientists so that I include 2-3 scientists per course topic in lecture. I designed a PowerPoint slide for each scientist that includes a picture of the scientist and how the research they have done that is related to that topic. Sometimes I include a screenshot of an abstract that I think students may be interested in reading. I do not typically include personal information in text on the slide, but students can glean some information from their photo, and I include a link to a website for students to read more about the scientist (see examples in Figure 1). I incorporated each slide into the particular part of the lecture in which their research focus fit best with the topic. For example, when introducing a scientist studying how mutations in voltage-gated Na^+^ channels relate to seizures, I discuss this right after we cover how voltage-gated Na^+^ channels contribute to action potentials. When I reach that particular slide in lecture, I discuss a few details of the scientist’s personal background, brief information about their research, and how their research connects with our current topic (approximately 2-5 minutes total for each scientist). I only include personal details about scientists that they have shared publicly, such as through the 500 Queer Scientists or Black in Neuro websites, or details that I have been given permission to share from people I know personally. In class, I announce that students will not be responsible for details of these scientists or their research on exams, but I often write extra credit exam questions or open-note assignment questions inspired by their research.

### Assessing student perceptions

I assessed student perceptions of these scientist highlights in three sections of a 400-level Neurobiology course in Spring and Fall 2022. This course is a requirement for neuroscience majors but also counts as an elective for biology majors and minors. I gave students a stand-alone anonymous survey at the end of the semester that asked for demographic information as well as their perceptions about my addition of diverse scientists to the course. I made it clear that students could skip any questions and gave “prefer not to answer” as an option on each demographic question. A total of 55 students completed the survey (see Table 1 for student demographics).

**Table 1.** Student self-identified demographics in a post-semester anonymous survey.

Race	53% White, 38% Asian, 19% Black/African American
Ethnicity	8% Hispanic/Latinx, 92% non-Hispanic/Latinx
Gender	65% Women, 31% Men, 4% genderqueer/non-binary
LGBTQ+	23% LGBTQ+, 77% not LGBTQ+
Disability	13% have a disability (self-identified), 87% do not
First-generation	33% First-generation college-student, 67% not first-generation
Under-represented	63% under-represented in science (self-identified), 37% not under-represented
Major	67% neuroscience, 29% biology, 4% psychology

I asked eight questions on a 5-point Likert scale (see Table 2). There were also three open-ended questions:

1. List one or more thing(s) that you learned from being introduced to neuroscientists throughout lectures in this course.
2. Do you have suggestions for improving this aspect of the class?
3. Are there any other comments related to this aspect of the class that you would like to make?

**Table 2.** Student perceptions of learning about diverse neuroscientists on Likert-scale questions (percentage of students responding with each answer).

	Strongly Agree	Agree	Neutral	Disagree	Strongly Disagree
I appreciated learning about a diverse group of neuroscientists this semester.	70.9%	23.6%	3.6%	1.8%	0%
Learning about neuroscientists was a waste of class time.	0%	5.5%	5.5%	36.4%	52.7%
I am more aware of the diversity of neuroscience researchers as a result of this class.	80.0%	18.2%	0%	1.8%	0%
Learning about neuroscientists made me feel more represented in neuroscience/science.	40.0%	23.6%	34.5%	0%	1.8%
Learning about neuroscientists helped me to do better in this class.	10.9%	23.6%	45.5%	16.4%	3.6%
Learning about neuroscientists and their research made me more interested in neuroscience.	61.8%	23.6%	7.3%	7.3%	0%
I am more aware of career options in neuroscience/science as a result of learning about neuroscientists in this class.	40.0%	45.5%	9.1%	3.6%	1.8%
Learning about neuroscientists has made me more likely to pursue a career that involves neuroscience in some way.	32.7%	21.8%	27.3%	16.4%	1.8%

In addition to overall reactions to this aspect of the class, I was also interested in whether this would impact students who self-reported that they belonged to one or more groups that were historically under-represented in the sciences more than other students. I used a chi-squared test on each of the eight Likert scale questions to determine if under-represented students responded differently than those who did not identify as belonging to one of these groups. Because of the extremely low frequency of “Strongly Disagree” or “Disagree” responses (0-2 students) on five out of the eight questions (see Table 2), I combined these two categories of responses for these five questions to perform the analyses. For questions in which there were still ≤ 1 response for all groups, I merged “Strongly Disagree,” “Disagree,” and “Neutral” together. For the second question about this aspect of the class being a waste of time, the opposite applied; there were no “Strongly Agree” responses, so this category was combined with “Agree.” When frequencies were still below five when combining these response categories, a Fisher’s Exact Test was used instead of a chi-squared test. I applied a Bonferroni correction to account for these eight analyses, such that α = 0.00625. When there was a significant difference between groups, Cramer’s V was used as a measure of effect size.

## Results

**Figure 2.**
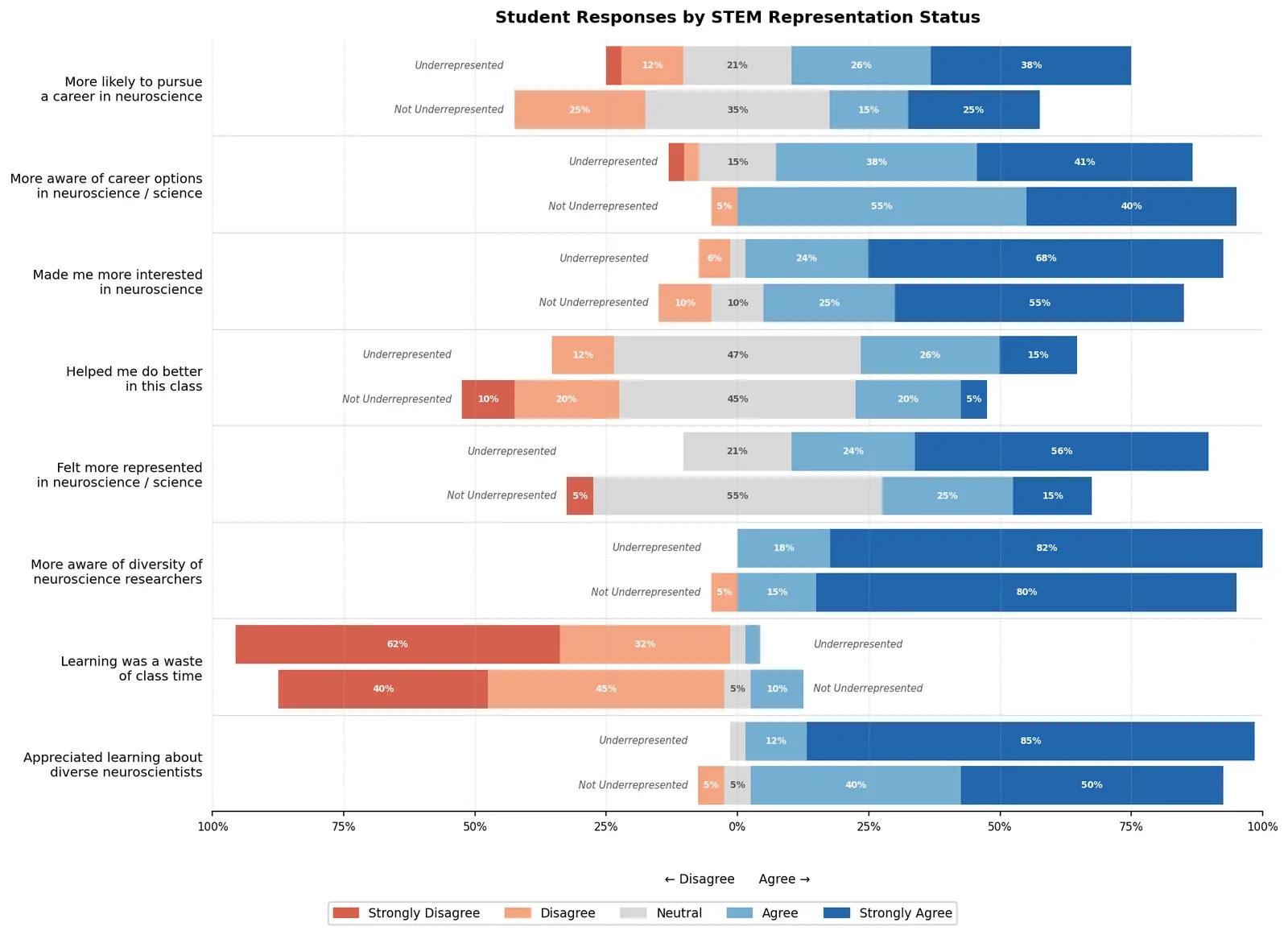
Student responses to eight course survey items comparing underrepresented (n = 34) and not underrepresented (n = 20) students in STEM (see Methods). For each item, the upper bar represents underrepresented students and the lower bar represents not underrepresented students. Bars extend leftward for disagreement and rightward for agreement, with neutral responses centered at zero.

Student demographic responses and responses to the Likert scale questions were similar across all three sections of the course. Of the students that responded to each demographic question, the percentage of each response is summarized in the supplementary tables. When asked about how they perceived the inclusion of diverse neuroscientists in the course, almost all students appreciated learning about diverse neuroscientists (94%; Table 2), and the vast majority (89%) disagreed or strongly disagreed that it was a waste of class time (Table 2). When responses to these two questions were separated by demographic categories, at least 50 (and up to 94.4) percent of students in every demographic group strongly agreed that they appreciated learning about neuroscientists, and at least 38.9 (up to 71.4) percent of each category of students strongly disagreed that it was a waste of time (Figure 2).

Almost all students (98%) reported that they were more aware of the diversity of neuroscience researchers, and 64% percent said that they felt more represented in neuroscience/science as a result of learning about these neuroscientists. The increase in diversity awareness was similar across demographic categories, with at least two-thirds of students strongly agreeing about this increase in awareness across groups. Interestingly, there was a significant difference in responses about the sense of representation between students who self-identified as belonging to a group historically under-represented in science and those who didn’t (*χ*^2^(2) = 10.74, *p* = 0.0047, Cramer’s V = 0.45), with 88% of under-represented students reporting an increase in representation and only 40% of other students reporting an increase (Figure 2). There were no other questions yielding a significant difference between these two groups.

There were mixed responses about whether learning about neuroscientists helped students do better in the class, with only 33% reporting that this aspect of the course improved their performance (Figure 2). For all demographic categories, the neutral response was most popular. However, most students (85%) reported more interest in neuroscience as a result of learning about diverse neuroscientists. The only category that had fewer than half of students strongly agreeing to this question was the group of students majoring in something other than neuroscience, but even this group still had 72.2% of students agreeing or strongly agreeing that they became more interested in the field. Furthermore, 85% of students felt more aware of career options in neuroscience/science, and 54% of students agreed that they are more likely to pursue a career that involves neuroscience in some way as a result of being introduced to diverse neuroscientists (Figure 2). In every demographic category, at least two-thirds of students said that they agreed or strongly agreed that their awareness of career options increased. Interestingly, 85.7% of students with a disability agreed or strongly agreed that they were more likely to pursue a career in neuroscience, with 64.8% of neuroscience majors also responding this way.

Almost all students took the time to answer the open-ended questions in the survey. In particular, when asked to identify what they learned from this aspect of the class, their responses tended to fall into one of six categories: recognizing the breadth of neuroscience, understanding that neuroscientists are diverse in many ways, feeling more represented in science, understanding that scientists are real people, appreciating the process of science, and feeling empowered to pursue science or neuroscience. See Table 3 for representative responses on this question.

**Table 3.** Representative student comments, by theme, about what they learned from being introduced to diverse neuroscientists.

**Breadth of neuroscience as a field:** “How vast and diverse the field of neurobiology is.” “There is also a large field of specialties within neuroscience and there is even more to be discerned.” “I learned about the complexity of neuroscience.” “The main learning point for me was seeing how broad the field of neuroscience is.”
**Recognizing diversity in neuroscientists:** “The diversity in science and the various backgrounds neuroscientists have overcome to be where they are.” “It is a diverse field. Researchers aren't typical old white men and can often being doing research when they're in danger too!” “The different factors that go into your work being accepted in the scientific community. It is not as simple as having good work. People will always look to question your work and your biases.” “The importance of diversity and representation in science. Importance of collaboration for the advancement of science. Biases associated with marginalized populations don't really apply to their scientific abilities!”
**Seeing people like them represented in the field:** “Most importantly there is a lot of diversity in this area of interest and that there are people who are like me/look like me/have similar cultural backgrounds in this field.” “Including different researchers made me feel more confident and comfortable in the class.”
**Scientists are real people:** “They are real people with real lives and work hard for what they know.” “The stories made the scientists more interesting as people rather than just authoritative figures.” “I enjoyed that you knew a majority of the neuroscientists and could tell a story about them.” “Peoples’ personal experiences outside of STEM can enhance their research.”
**Understanding the process of science:** “Neuroscientists are actually studying some of the questions I've asked in my life and thought I was stupid for not knowing the answer.” “My biggest take away was seeing how we come to know/learn things. In other words, I was able to learn content and also have exposure to the scientists doing the work. It made the content more memorable and more interesting, and I liked being able to know where the info I was learning originated.” “I learned that the material we are learning is still being carefully investigated by neuroscientists, highlighting the ever-changing and ever-growing field.” “I learned the real-life application of what we were focusing on in lecture. I learned the practical logistics behind researching those topics. I learned about the way the scientific community accepts/rejects different studies, and how that can be influenced by timing, other on-going research, as well as personal factors/discrimination”
**Feeling more empowered to pursue neuroscience:** “I have been looking at their research papers as I decide on PhD topic” “I thought it was helpful and interesting to know how I could participate in doing research because Dr. Northcutt knows some of these other neuroscientists” “The opportunities there are and various areas to work in neuroscience.”

Students also had helpful suggestions for improving this aspect of the class. The most common suggestion was to incorporate additional assignments that build off the highlighted neuroscientists’ work, including assigning papers based on their work, discussing the scientists’ papers, adding short out-of-class assignments, and writing extra-credit exam questions about the scientists. Other students said that I should provide even more information about each scientist, either through short videos or by giving additional context myself. Finally, several students thought that I should focus even more on younger scientists, early in their careers, that the students can better identify with.

## Discussion

Based on feedback I received from my students, briefly introducing diverse scientists during lectures was a productive use of class time, helping many students feel more represented in class, expanding students’ awareness of neuroscience-related career options, and even increasing their interest in pursuing one of these careers. Approximately a third of students also felt that learning about these scientists helped them succeed in the course, likely due to their increased sense of representation in science. Indeed, increases in in the perception of representation and belonging are associated with better performance in introductory biology, chemistry, and engineering courses (Fink et al., 2020; Schar et al., 2017; Schinske et al., 2016). This increased perception of representation was particularly impactful for students who see themselves as historically under-represented in the sciences, similar to the finding that Scientist Spotlight assignments in a biology course had the most impact on scientist relatability for females and first-generation students (Metzger et al., 2023). Not only did students see themselves represented, but they also commented on their increased appreciation of the diversity of neuroscience as a field (in terms of sub-disciplines) and their better understanding of the process of science.

Although I did not specifically assess the impact of including younger or early-career scientists in my examples, student comments revealed that they related most to these scientists and would like me to include more, as I have done since collecting these data. Given that stereotypes of scientists often include that they are middle-aged or elderly (Ferguson & Lezotte, 2020; Sheffield et al., 2021), and textbooks often include late-career scientists as examples, exposure to a diversity of ages/stages is helpful for students. Student interviews and discussions have previously been shown to disrupt some of these stereotypes (Sheffield et al., 2021), but building examples into class lectures is more approachable for many instructors. This aspect of diversity may be even more important at primarily undergraduate institutions, where there are no graduate students or postdoctoral fellows for undergraduates to interact with. However, even at large research institutions, these interactions are not common for most undergraduates.

One advantage of including diverse scientists during lecture is that it takes very little class time and can be employed in a wide variety of classes. It is also very flexible, as you could expand on what is done in class to give students assignments related to these scientists. For example, students could read a paper written by each scientist and write a short summary about how their research relates to the current class topic. Project DiViNe (Frenzel et al., 2022) and Scientist Spotlights (scientistspotlights.org) are useful tools for these teaching resources. Students could also find their own scientists that they identify with, especially if you provided them with some of these online resources. The process of writing similar biographies has previously been shown to increase science identity both for the authors and for other students reading them (Aranda et al., 2021). An assignment comparing and contrasting career choices would also be interesting, and some scientists might also be willing to answer questions via email or have a short zoom meeting to discuss their career trajectory and decision-making process. Given that active learning also increases students’ sense of belong, particularly for historically marginalized groups (Harry et al., 2024), these types of assignments may make this intervention even more impactful. I typically ask students to write two literature review papers throughout the semester, and I give them a choice of topics. After receiving feedback from students asking me to expand on the diverse neuroscientists intro, I now have at least several paper topic options that build from scientists we discussed in class; these are often the most popular topics.

I was surprised to learn that students particularly identified with scientists that I know personally, and I now include more of these examples. Sometimes I know these people well (good friends from graduate school or former research students, for example), and some of these people I only see occasionally at conferences. I even tell students fun anecdotes about scientists I do not know personally; for example, I tell them about an email exchange I once had with Dr. Eric Kandel and about helping him find his book signing table at a Society for Neuroscience meeting one year. These personal stories seem to be the most powerful in terms of making the scientists seem relatable. The reason that I have not included a long list of scientists that could be inserted into a similar course is that the personal experiences seemed so important that I encourage anyone using this approach to include people they know personally. Furthermore, Costello et al. (2025) noted that highlighting diverse scientists in a course can facilitate relationship building between students and faculty; although I did not directly assess this, I have noticed that students seem more eager to come to office hours and engage with me outside of class in this course compared to courses in which I have not yet incorporated a diverse scientist component. The personal anecdotes likely play a role in this relationship building.

When attempting to increase diversity and inclusion within a class, it is particularly important to clearly state the goals to students. Harry et al. (2024) discuss that even professors who do not belong to marginalized groups can improve students’ belonging by acknowledging and reflecting on their own identities and by incorporating examples of diversity. I did this by discussing my goals on the first day of class, as I highlighted aspects of the syllabus, and then I reminded students as I discussed the first scientist during our first lecture topic of the semester. However, there are other ways this could be accomplished, depending on the size and format of the class.

Although there are great resources available, one challenge is identifying scientists that truly represent the wide array of scientists and their identities who have been willing to publicly share their experiences. What I have struggled with most is including disabled neuroscientists. Many disabilities are hidden, and I do not want to mention a disability that someone has not discussed publicly themselves. I have been able to include several examples of scientists I know personally who have given me permission to discuss their disabilities (and how it has impacted their career/science) freely with my students, but I am not aware of public databases that disabled scientists have helped to create.

In conclusion, briefly highlighting a diverse array of neuroscientists during lectures is a meaningful way to increase students’ sense of representation and identity in science, and students overwhelmingly appreciate these scientist introductions. Including scientists you know personally and who are just beginning their career is important for student relatability. This technique adds an additional option for instructors to consider as they work to foster the development of scientific identities and belonging in neuroscience students.

### Address correspondence to:

Katharine Northcutt, Biology and Neuroscience, Mercer University. Email: northcutt_kv@mercer.edu.

## Supplementary Material

Supplementary Tables
