# Supplementary material for: Fostering Belonging Through Introducing Diverse Scientists in Undergraduate Neuroscience Lectures
Source: J Undergrad Neurosci Educ. 2026 Jun 23;24(2):61–70. doi: 10.59390/001c.161078 (PMC13625901; doi:10.59390/001c.161078)
Supplement: Supplementary file 1 [file junejournal_2026_24_2_161078_341146.docx]

**Supplementary Tables**

*Table 1. Progression of course topics throughout a typical semester.*

| Week of the course | Topic |
| --- | --- |
| Week 1 | Cellular properties of neurons |
| Week 2 | Action potentials |
| Week 3 | Synaptic Transmission |
| Week 4 | Neurotransmitters |
| Week 5 | Neuroanatomy |
| Week 6 | Chemosenses (olfaction and gustation) |
| Week 7 | Somatosensation |
| Weeks 8-9 | Vision |
| Weeks 10-11 | Motor systems and movement |
| Week 12 | Neural development |
| Week 13 | Synaptic plasticity |
| Week 14 | Learning and memory |
| Week 15 | Autonomic nervous system and stress |
| Week 16 | Sex differences in the nervous system |

|  | | Strongly Agree | Agree | Neutral | Disagree | Strongly Disagree |
| --- | --- | --- | --- | --- | --- | --- |
| All responses (55) | | 70.9% | 23.6% | 3.6% | 1.8% | 0% |
| Under-represented in STEM | Under-represented (34) | 85.3% | 11.7% | 2.9% | 0% | 0% |
|  | Not under-represented (20) | 50.0% | 40.0% | 5.0% | 5.0% | 0% |
| Race | White (28) | 67.9% | 28.6% | 3.6% | 0% | 0% |
|  | All other responses (26) | 77.0% | 15.4% | 3.8% | 3.8% | 0% |
| Ethnicity | Hispanic/Latinx (4) | 75.0% | 25.0% | 0% | 0% | 0% |
|  | Not Hispanic/Latinx (49) | 73.5% | 20.4% | 4.1% | 2.0% | 0% |
| Gender | Woman (35) or genderqueer/  nonbinary (2) | 73.0% | 24.3% | 2.7% | 0% | 0% |
|  | Man (17) | 70.6% | 17.6% | 5.9% | 5.9% | 0% |
| LTBTQ+ | LGBTQ+ (12) | 91.7% | 8.4% | 0% | 0% | 0% |
|  | Not LGBTQ+ (41) | 68.3% | 24.4% | 4.9% | 2.4% | 0% |
| Disability | Disability (7) | 85.7% | 14.3% | 0% | 0% | 0% |
|  | No disability (46) | 71.7% | 21.7% | 4.3% | 2.2% | 0% |
| First-generation college student | First-gen (18) | 94.4% | 0% | 5.6% | 0% | 0% |
|  | Not first-gen (36) | 61.1% | 33.3% | 2.8% | 2.8% | 0% |
| Major | Neuroscience (37) | 75.7% | 18.9% | 2.7% | 2.7% | 0% |
|  | Other major (18) | 61.1% | 33.3% | 5.6% | 0% | 0% |

*Table 2.* Student responses to “I appreciated learning about a diverse group of neuroscientists this semester.”

*Note:* The number of students responding to this question belonging to each demographic group is in parentheses.

*Table 3.* Student responses to “Learning about neuroscientists was a waste of class time.”

|  | | Strongly Agree | Agree | Neutral | Disagree | Strongly Disagree |
| --- | --- | --- | --- | --- | --- | --- |
| All responses (55) | | 0% | 5.5% | 5.5% | 36.4% | 52.7% |
| Under-represented in STEM | Under-represented (34) | 0% | 2.9% | 2.9% | 32.4% | 61.8% |
|  | Not under-represented (20) | 0% | 10.0% | 5.0% | 45.0% | 40% |
| Race | White (28) | 0% | 3.6% | 0% | 4.6% | 50.0% |
|  | All other responses (26) | 0% | 7.7% | 7.7% | 26.9% | 57.7% |
| Ethnicity | Hispanic/Latinx (4) | 0% | 0% | 0% | 50.0% | 50.0% |
|  | Not Hispanic/Latinx (49) | 0% | 6.1% | 4.1% | 34.7% | 55.1% |
| Gender | Woman (35) or genderqueer/  nonbinary (2) | 0% | 2.7% | 5.4% | 35.1% | 56.8% |
|  | Man (17) | 0% | 11.7% | 0% | 41.2% | 47.1% |
| LTBTQ+ | LGBTQ+ (12) | 0% | 0% | 8.3% | 33.3% | 58.3% |
|  | Not LGBTQ+ (41) | 0% | 7.3% | 2.4% | 36.6% | 53.7% |
| Disability | Disability (7) | 0% | 0% | 0% | 28.6% | 71.4% |
|  | No disability (46) | 0% | 6.5% | 4.3% | 37.0% | 52.2% |
| First-generation college student | First-gen (18) | 0% | 5.6% | 5.6% | 27.8% | 61.1% |
|  | Not first-gen (36) | 0% | 5.6% | 5.6% | 38.9% | 50.0% |
| Major | Neuroscience (37) | 0% | 5.4% | 5.4% | 29.7% | 59.5% |
|  | Other major (18) | 0% | 5.6% | 5.6% | 50.0% | 38.9% |

*Note:* The number of students responding to this question belonging to each demographic group is in parentheses.

*Table 4.* Student responses to “I am more aware of the diversity of neuroscience researchers as a result of this class.”

|  | | Strongly Agree | Agree | Neutral | Disagree | Strongly Disagree |
| --- | --- | --- | --- | --- | --- | --- |
| All responses (55) | | 80.0% | 18.2% | 0% | 1.8% | 0% |
| Under-represented in STEM | Under-represented (34) | 82.4% | 17.6% | 0% | 0% | 0% |
|  | Not under-represented (20) | 80.0% | 15.0% | 0% | 5.0% | 0% |
| Race | White (28) | 78.6% | 21.4% | 0% | 0% | 0% |
|  | All other responses (26) | 84.6% | 11.5% | 0% | 3.8% | 0% |
| Ethnicity | Hispanic/Latinx (4) | 75.0% | 25.0% | 0% | 0% | 0% |
|  | Not Hispanic/Latinx (49) | 83.7% | 14.3% | 0% | 2.0% | 0% |
| Gender | Woman (35) or genderqueer/  nonbinary (2) | 78.4% | 21.6% | 0% | 0% | 0% |
|  | Man (17) | 88.2% | 5.9% | 0% | 5.9% | 0% |
| LTBTQ+ | LGBTQ+ (12) | 83.3% | 16.7% | 0% | 0% | 0% |
|  | Not LGBTQ+ (41) | 82.9% | 14.6% | 0% | 2.4% | 0% |
| Disability | Disability (7) | 85.7% | 14.3% | 0% | 0% | 0% |
|  | No disability (46) | 82.6% | 15.2% | % | 2.2% | 0% |
| First-generation college student | First-gen (18) | 94.4% | 5.6% | 0% | 0% | 0% |
|  | Not first-gen (36) | 75.0% | 22.2% | 0% | 2.8% | 0% |
| Major | Neuroscience (37) | 86.5% | 10.8% | 0% | 2.7% | 0% |
|  | Other major (18) | 66.7% | 33.3% | 0% | 0% | 0% |

*Note:* The number of students responding to this question belonging to each demographic group is in parentheses.

|  | | Strongly Agree | Agree | Neutral | Disagree | Strongly Disagree |
| --- | --- | --- | --- | --- | --- | --- |
| All responses (55) | | 40.0% | 23.6% | 34.5% | 0% | 1.8% |
| Under-represented in STEM | Under-represented (34) | 55.9% | 23.5% | 20.6% | 0% | 0% |
|  | Not under-represented (20) | 15.0% | 25.0% | 55.0% | 0% | 5.0% |
| Race | White (28) | 35.7% | 17.9% | 46.4% | 0% | 0% |
|  | All other responses (26) | 46.2% | 30.8% | 19.2% | 0% | 3.8% |
| Ethnicity | Hispanic/Latinx (4) | 25.0% | 25.0% | 50.0% | 0% | 0% |
|  | Not Hispanic/Latinx (49) | 42.9% | 24.5% | 30.6% | 0% | 2.0% |
| Gender | Woman (35) or genderqueer/  nonbinary (2) | 51.4% | 21.6% | 27.0% | 0% | 0% |
|  | Man (17) | 17.6% | 29.4% | 47.1% | 0% | 5.9% |
| LTBTQ+ | LGBTQ+ (12) | 66.7% | 16.7% | 16.7% | 0% | 0% |
|  | Not LGBTQ+ (41) | 34.1% | 26.8% | 36.6% | 0% | 2.4% |
| Disability | Disability (7) | 71.4% | 14.3% | 14.3% | 0% | 0% |
|  | No disability (46) | 37.0% | 26.1% | 34.8% | 0% | 2.2% |
| First-generation college student | First-gen (18) | 50.0% | 27.8% | 22.2% | 0% | 0% |
|  | Not first-gen (36) | 36.1% | 22.2% | 38.9% | 0% | 2.8% |
| Major | Neuroscience (37) | 51.4% | 18.9% | 27.0% | 0% | 2.7% |
|  | Other major (18) | 16.7% | 33.3% | 50.0% | 0% | 0% |

*Table 5.* Student responses to “Learning about neuroscientists made me feel more represented in neuroscience/science.”

*Note:* The number of students responding to this question belonging to each demographic group is in parentheses.

*Table 6.* Student responses to “Learning about neuroscientists helped me to do better in this class.”

|  | | Strongly Agree | Agree | Neutral | Disagree | Strongly Disagree |
| --- | --- | --- | --- | --- | --- | --- |
| All responses (55) | | 10.9% | 23.6% | 45.5% | 16.4% | 3.6% |
| Under-represented in STEM | Under-represented (34) | 14.7% | 26.4% | 47.1% | 11.8% | 0% |
|  | Not under-represented (20) | 5.0% | 20.0% | 45.0% | 20.0% | 10.0% |
| Race | White (28) | 7.1% | 28.6% | 50.0% | 10.7% | 3.6% |
|  | All other responses (26) | 15.4% | 19.2% | 42.3% | 19.2% | 3.9% |
| Ethnicity | Hispanic/Latinx (4) | 0% | 25.0% | 75.0% | 0% | 0% |
|  | Not Hispanic/Latinx (49) | 12.2% | 24.5% | 44.9% | 14.3% | 4.1% |
| Gender | Woman (35) or genderqueer/  nonbinary (2) | 10.8% | 24.3% | 45.9% | 18.9% | 0% |
|  | Man (17) | 11.8% | 23.5% | 47.1% | 5.9% | 11.7% |
| LTBTQ+ | LGBTQ+ (12) | 33.3% | 25.0% | 41.7% | 0% | 0% |
|  | Not LGBTQ+ (41) | 4.9% | 24.4% | 48.8% | 17.1% | 4.9% |
| Disability | Disability (7) | 14.3% | 28.6% | 57.1% | 0% | 0% |
|  | No disability (46) | 10.9% | 23.9% | 45.7% | 15.2% | 4.3% |
| First-generation college student | First-gen (18) | 16.7% | 27.8% | 44.4% | 11.1% | 0% |
|  | Not first-gen (36) | 8.3% | 22.2% | 47.2% | 16.7% | 5.6% |
| Major | Neuroscience (37) | 16.2% | 27.0% | 37.8% | 13.5% | 5.4% |
|  | Other major (18) | 0% | 16.7% | 61.1% | 22.2% | 0% |

*Note:* The number of students responding to this question belonging to each demographic group is in parentheses.

*Table 7.* Student responses to “Learning about neuroscientists and their research made me more interested in neuroscience.”

|  | | Strongly Agree | Agree | Neutral | Disagree | Strongly Disagree |
| --- | --- | --- | --- | --- | --- | --- |
| All responses (55) | | 61.8% | 23.6% | 7.3% | 7.3% | 0% |
| Under-represented in STEM | Under-represented (34) | 67.6% | 23.5% | 2.9% | 5.9% | 0% |
|  | Not under-represented (20) | 55.0% | 25.0% | 10.0% | 10.0% | 0% |
| Race | White (28) | 60.7% | 28.6% | 7.1% | 3.6% | 0% |
|  | All other responses (26) | 65.4% | 19.2% | 3.8% | 11.5% | 0% |
| Ethnicity | Hispanic/Latinx (4) | 50.0% | 50.0% | 0% | 0% | 0% |
|  | Not Hispanic/Latinx (49) | 65.3% | 22.4% | 6.1% | 6.1% | 0% |
| Gender | Woman (35) or genderqueer/  nonbinary (2) | 64.9% | 24.3% | 5.4% | 5.4% | 0% |
|  | Man (17) | 58.8% | 23.5% | 5.8% | 11.8% | 0% |
| LTBTQ+ | LGBTQ+ (12) | 75.0% | 16.7% | 8.3% | 0% | 0% |
|  | Not LGBTQ+ (41) | 61.0% | 26.8% | 4.9% | 7.3% | 0% |
| Disability | Disability (7) | 85.7% | 0% | 14.3% | 0% | 0% |
|  | No disability (46) | 60.9% | 28.2% | 4.3% | 6.5% | 0% |
| First-generation college student | First-gen (18) | 83.3% | 11.1% | 0% | 5.6% | 0% |
|  | Not first-gen (36) | 52.8% | 30.6% | 11.1% | 5.6% | 0% |
| Major | Neuroscience (37) | 70.3% | 21.6% | 2.7% | 5.4% | 0% |
|  | Other major (18) | 44.4% | 27.8% | 16.7% | 11.1% | 0% |

*Note:* The number of students responding to this question belonging to each demographic group is in parentheses.

*Table 8.* Student responses to “I am more aware of career options in neuroscience/science as a result of learning about neuroscientists in this class.”

|  | | Strongly Agree | Agree | Neutral | Disagree | Strongly Disagree |
| --- | --- | --- | --- | --- | --- | --- |
| All responses (55) | | 40.0% | 45.5% | 9.1% | 3.6% | 1.8% |
| Under-represented in STEM | Under-represented (34) | 41.1% | 38.2% | 14.7% | 2.9% | 2.9% |
|  | Not under-represented (20) | 40.0% | 55.0% | 0% | 5.0% | 0% |
| Race | White (28) | 42.9% | 46.4% | 10.7% | 0% | 0% |
|  | All other responses (26) | 38.5% | 42.3% | 7.7% | 7.7% | 3.8% |
| Ethnicity | Hispanic/Latinx (4) | 25.0% | 50.0% | 25.0% | 0% | 0% |
|  | Not Hispanic/Latinx (49) | 42.9% | 44.9% | 6.1% | 4.1% | 2.0% |
| Gender | Woman (35) or genderqueer/  nonbinary (2) | 45.9% | 40.5% | 10.8% | 2.7% | 0% |
|  | Man (17) | 29.4% | 52.9% | 5.8% | 5.8% | 5.8% |
| LTBTQ+ | LGBTQ+ (12) | 41.7% | 41.7% | 16.7% | 0% | 0% |
|  | Not LGBTQ+ (41) | 41.4% | 46.3% | 4.8% | 4.8% | 2.4% |
| Disability | Disability (7) | 42.9% | 42.9% | 14.3% | 0% | 0% |
|  | No disability (46) | 41.3% | 45.6% | 6.5% | 4.3% | 2.2% |
| First-generation college student | First-gen (18) | 50.0% | 38.9% | 0% | 5.6% | 5.6% |
|  | Not first-gen (36) | 36.1% | 50.0% | 11.1% | 2.8% | 0% |
| Major | Neuroscience (37) | 51.4% | 35.1% | 8.1% | 2.7% | 2.7% |
|  | Other major (18) | 16.7% | 50.0% | 11.1% | 2.8% | 0% |

*Note:* The number of students responding to this question belonging to each demographic group is in parentheses.

*Table 9.* Student responses to “Learning about neuroscientists has made me more likely to pursue a career that involves neuroscience in some way.”

|  | | Strongly Agree | Agree | Neutral | Disagree | Strongly Disagree |
| --- | --- | --- | --- | --- | --- | --- |
| All responses (55) | | 32.7% | 21.8% | 27.3% | 16.4% | 1.8% |
| Under-represented in STEM | Under-represented (34) | 38.2% | 26.5% | 20.6% | 11.8% | 2.9% |
|  | Not under-represented (20) | 25.0% | 15.0% | 35.0% | 25.0% | 0% |
| Race | White (28) | 28.6% | 25.0% | 17.9% | 28.6% | 0% |
|  | All other responses (26) | 38.5% | 19.2% | 34.6% | 3.8% | 3.8% |
| Ethnicity | Hispanic/Latinx (4) | 25.0% | 25.0% | 0% | 50.0% | 0% |
|  | Not Hispanic/Latinx (49) | 34.7% | 22.4% | 28.6% | 12.2% | 2.0% |
| Gender | Woman (35) or genderqueer/  nonbinary (2) | 35.1% | 24.3% | 21.6% | 16.2% | 2.7% |
|  | Man (17) | 29.4% | 17.6% | 35.3% | 17.6% | 0% |
| LTBTQ+ | LGBTQ+ (12) | 33.3% | 33.3% | 25.0% | 8.3% | 0% |
|  | Not LGBTQ+ (41) | 34.1% | 19.5% | 26.8% | 17.1% | 2.4% |
| Disability | Disability (7) | 71.4% | 14.3% | 0% | 14.3% | 0% |
|  | No disability (46) | 28.3% | 23.9% | 30.4% | 15.2% | 2.2% |
| First-generation college student | First-gen (18) | 38.9% | 22.2% | 27.8% | 5.6% | 5.6% |
|  | Not first-gen (36) | 30.6% | 22.2% | 27.8% | 19.4% | 0% |
| Major | Neuroscience (37) | 43.2% | 21.6% | 27.0% | 8.1% | 0% |
|  | Other major (18) | 11.1% | 22.2% | 27.8% | 33.3% | 5.6% |

*Note:* The number of students responding to this question belonging to each demographic group is in parentheses.
